# Development of a convenient and effective hypertension risk prediction model and exploration of the relationship between Serum Ferritin and Hypertension Risk: a study based on NHANES 2017—March 2020

**DOI:** 10.3389/fcvm.2023.1224795

**Published:** 2023-09-06

**Authors:** Shuang Guo, Jiu-Xin Ge, Shan-Na Liu, Jia-Yu Zhou, Chang Li, Han-Jie Chen, Li Chen, Yu-Qiang Shen, Qing-Li Zhou

**Affiliations:** ^1^ Information Center, The Fourth Affiliated Hospital, Zhejiang University School of Medicine, Yiwu, China; ^2^ Department of Cardiology, The Fourth Affiliated Hospital, Zhejiang University School of Medicine, Yiwu, China; ^3^ Xinjiang Second Medical College, Karamay, China

**Keywords:** machine learning, hypertension, risk prediction, stacking, serum ferritin, trend analysis

## Abstract

**Background:**

Hypertension is a major public health problem, and its resulting other cardiovascular diseases are the leading cause of death worldwide. In this study, we constructed a convenient and high-performance hypertension risk prediction model to assist in clinical diagnosis and explore other important influencing factors.

**Methods:**

We included 8,073 people from NHANES (2017—March 2020), using their 120 features to form the original dataset. After data pre-processing, we removed several redundant features through LASSO regression and correlation analysis. Thirteen commonly used machine learning methods were used to construct prediction models, and then, the methods with better performance were coupled with recursive feature elimination to determine the optimal feature subset. After data balancing through SMOTE, we integrated these better-performing learners to construct a fusion model based for predicting hypertension risk on stacking strategy. In addition, to explore the relationship between serum ferritin and the risk of hypertension, we performed a univariate analysis and divided it into four level groups (Q1 to Q4) by quartiles, with the lowest level group (Q1) as the reference, and performed multiple logistic regression analysis and trend analysis.

**Results:**

The optimal feature subsets were: age, BMI, waist, SBP, DBP, Cre, UACR, serum ferritin, HbA1C, and doctors recommend reducing salt intake. Compared to other machine learning models, the constructed fusion model showed better predictive performance with precision, accuracy, recall, F1 value and AUC of 0.871, 0.873, 0.871, 0.869 and 0.966, respectively. For the analysis of the relationship between serum ferritin and hypertension, after controlling for all co-variates, OR and 95% CI from Q2 to Q4, compared to Q1, were 1.396 (1.176–1.658), 1.499 (1.254–1.791), and 1.645 (1.360–1.989), respectively, with *P* < 0.01 and *P* for trend <0.001.

**Conclusion:**

The hypertension risk prediction model developed in this study is efficient in predicting hypertension with only 10 low-cost and easily accessible features, which is cost-effective in assisting clinical diagnosis. We also found a trend correlation between serum ferritin levels and the risk of hypertension.

## 1. Background

Hypertension is characterized by a sustained increase in systemic arterial blood pressure and might be associated with functional and/or structural damage to the heart, brain, and kidneys (1). Studies have shown that by 2025, approximately one-third of the global population is expected to suffer from hypertension (2), accounting for approximately 13% of total global mortality and 7% of total disability-adjusted life expectancy, substantially increasing the financial burden on patients and healthcare systems. Although the age-standardized prevalence of hypertension has decreased over the past decade, it is still increasing in low-income and middle-income countries (1). China is a prominently affected country, where 22.6%–33.6% of the total population is diagnosed with hypertension, causing an estimated 23 million deaths per year; the percentage of adults with high blood pressure is 23.2% (3). Unfortunately, the current awareness rate of hypertension in China is only 51.6% (4), and nearly 50% of the people do not know that they have hypertension. The low awareness rate seriously affects the follow-up treatment, resulting in an increase in the incidence and a delayed inflection point. Given this serious problem, early detection and control of hypertension might be a cost-effective way to reduce the burden of hypertension-related diseases (5).

The diagnosis of hypertension mainly includes (1) office blood pressure measurement (OBPM): a simple procedure but with a poor diagnosis effect for masked hypertension and white coat hypertension (6); (2) 24 h ambulatory blood pressure measurement (ABPM): the gold standard for diagnosis, but its accessibility is poor because it is expensive, uncomfortable for the patients, and other reasons (7); (3) home blood pressure measurement (HBPM): easy to operate, but due to non-standard measurement methods and random measurement time, the method is not very accurate; meanwhile, the home blood pressure monitor is poorly equipped in Chinese households (8–10). Therefore, a new method, which saves time and money and is comfortable for the patient, needs to be developed to assist in diagnosis.

Artificial intelligence has gained popularity recently, and machine learning techniques, which are an important part of artificial intelligence, are extremely effective in identifying hypertension risk factors and predicting the risk of the disease. By integrating big data resources and using machine learning methods to construct morbidity risk prediction models, early identification of people at high risk of hypertension is possible. Through early intervention, the development of hypertension can be prevented and delayed, and thus, the risk of cardiovascular diseases can be significantly decreased (11–13). New predictors of hypertension might also be identified, fully utilizing the massive medical data (14). This approach is efficient, comfortable for the patient, and cost-effective to some extent.

Several studies around the world have constructed hypertension risk prediction models. These studies used different machine learning methods and incorporated different factors for prediction, and all showed high performance of the models (4–6). However, they only focused on clinically accepted risk factors, such as age, obesity, family history, hyperglycemia, hyperlipidemia, smoking, alcohol consumption, etc (15–19), might ignore some important influencing factors. Also, most studies only used several commonly used machine learning methods for simple cross-sectional comparisons without further improving the performance of the model.

In this study, based on literature research and expert guidance, we incorporated more comprehensive information and extracted eligible subjects and their information from NHANES to develop a high-performance fusion model based on stacking to predict the risk of hypertension. In addition, serum ferritin (SF) was included in the best feature subset for the first time among many studies that predicted the risk of hypertension by machine learning methods; therefore, we further analyzed its association with hypertension and obtained a trend correlation between the risk of hypertension and SF levels.

## 2. Methods

### 2.1. Study population

The National Health and Nutrition Examination Survey (NHANES) is a population-based cross-sectional survey conducted by the National Center for Health Statistics (NCHS) to assess the health and nutrition status of adults and children in America (19–22). The NHANES uses a complex multistage sampling design to collect data from a representative sample of the population. The NHANES has been conducted regularly since the 1960s and provides valuable information on the health and nutrition of the U.S. population. We used the data from the official website of NHANES from 2017 to March 2020. More information on the NHANES can be found on the website http://www.CDC.gov/nchs/nhanes/.

**Figure 1:**
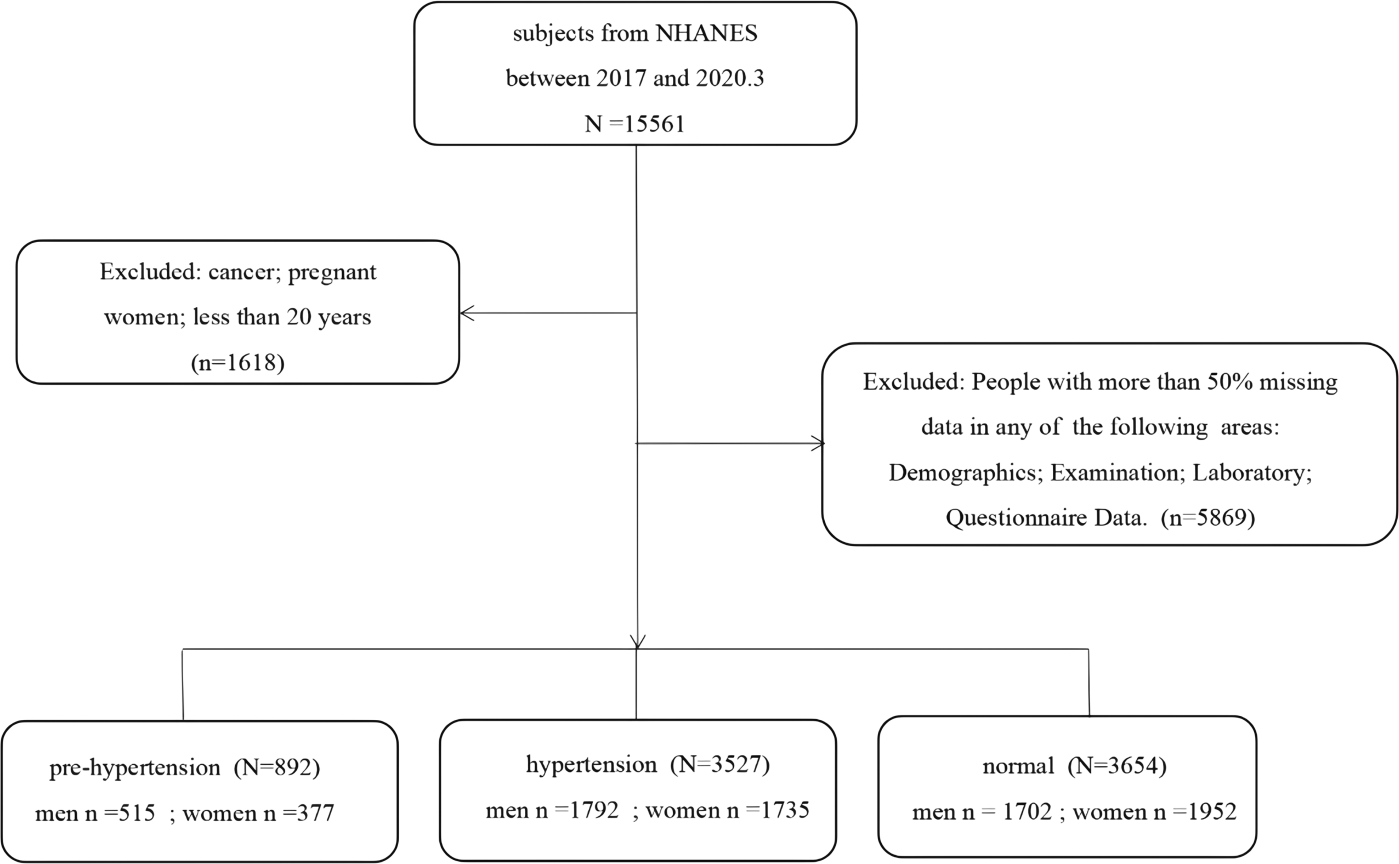
Flowchart of participant selection.

Based on literature review and the guidance of experts from the Department of Cardiovascular Medicine, we extracted information on 120 items from 37 original datasets from the four dimensions of demographics, body measurements, laboratory tests, and questionnaires contained in the NHANES database (23, 24). Data on pregnant women, cancer patients, and those under 20 years (*n* = 1,618) were excluded from the original dataset containing 15,561 participants. Then, the participants with a lot of missing information, such as missing more than 80% of any dimension of demographic, body measurement, laboratory test, or questionnaire were excluded (*n* = 5,869). Finally, data on 8,073 participants were included in the study. A flowchart concerning participant selection is shown in Figure 1.

### 2.2. Definition of hypertension

We classify patients with hypertension as hypertensive (0) if they have a history of taking medication for hypertension or a history of diagnosis. If there is no such condition, it can be divided by SBP/DBP (≥140 mmHg or ≥90 mmHg is “3” for hypertensive patients, 130–139 mmHg or 80–89 mmHg is “2” for high blood pressure, <130 mmHg and <80 mmHg is “1” for normal people).

### 2.3. Missing value processing

After obtaining the original dataset, mode filling and *K* nearest neighbors filling were used for categorical variables and continuous variables, respectively.

Mode filling was achieved by replacing missing values with the plural (i.e., the value with the highest number of occurrences) of the feature. We used it for filling the categorical variables because it is easy to implement and provides satisfactory results in most cases.

*K* nearest neighbors filling is a similarity-based padding method. It fills the missing values by finding the nearest neighbors around missing values and using their average or weighted average. We used it to fill continuous variables because it accounts for the similarity between data points and can partly reduce bias.

### 2.4. Feature selection

In this study, we first removed redundant features through LASSO regression and correlation analysis, which included features that were not significantly associated with the disease and one of the two features that were highly correlated (correlation coefficient ≥0.7), and then used recursive feature elimination (RFE) to determine the optimal feature subset (25). Since RFE needs to be used along with a prediction model, we firstly conducted a preliminary selection of the models before extracting the features. Based on previous studies, we selected 13 representative methods (LR, SVC, KNN, NB, MLP, DT, ANN, DNN, RF, AB, XGB, LGBM, and ET) in the field of traditional machine learning, deep learning, and integrated learning to model the original data (6, 13, 18, 26–29). Then, the models with better performance were used in combination with RFE for feature selection. Finally, the optimal feature subset was determined. For the optimal feature subset, we also performed some visualization analysis, such as plotting feature ranking graphs and correlation heat maps.

Recursive Feature Elimination (RFE) is a commonly used feature selection method that selects features by recursively constructing models and gradually reducing the number of features (24, 25, 30). The workflow of RFE was as follows:

(1) All features were used to construct the model;
(2) The importance of each feature in the model was calculated;
(3) The least important features were removed;
(4) Steps 2 and 3 were repeated until the required number of features reached a predetermined value.

### 2.5. Data balance

For processing data balance, we selected the most commonly used SMOTE (Synthetic Minority Over-sampling Technique), which increases the number of samples in a minority category by synthesizing new samples (31–33). The workflow of the SMOTE algorithm was as follows.

(1) A sample of a minority category was randomly selected.
(2) The *K* nearest neighbors of that sample were found.
(3) The nearest neighbor was randomly selected, and interpolation was performed between the neighbor and the sample to generate a new sample.
(4) Steps 2 and 3 were repeated until the number of samples in the minority category reached a predetermined value.

The SMOTE can be easily implemented and can effectively balance datasets with unbalanced categories, but it creates some synthetic samples that might not exist in the original dataset. Therefore, to avoid producing more synthetic samples and ensure that our study was conducted efficiently, we performed the data balancing step after determining the best feature subset.

### 2.6. Model evaluation metrics

The prediction model constructed in this study is a multi-classification prediction, and to evaluate our model, five metrics were used: ACC (accuracy), precision, recall, F1 value, and AUC.

Since this study is a triple classification task and considering the unbalance samples, the Macro Average rule was used to weight these evaluation criteria. If the patients with hypertension are recorded as True (1), the rest are False (0) and ACC1, P1, and R1 are calculated; the people with high blood pressure are recorded as True (1), the rest are False (0) and then calculate ACC2, P2, and R2; if normal people are recorded as True (1), the rest are False (0) and ACC3, P3, and R3 are calculated. From this, the corresponding F1 values can also be calculated separately, a final value can be obtained by the weighted average of the corresponding indexes of each group separately, which can be used as the evaluation index of this research model.

In order to evaluate the model as more objective and accurate, 10-fold cross-validation was carried out for the final prediction model, that is, all samples in the data set were randomly divided into 10 mutually exclusive subsets with similar sizes and approximately the same number of events, and during each training round, 9 subsets were selected in turn to form the training set and the remaining subsets formed the test set. The model needs to run 10 times with different training and test sets, respectively, and the final result is the average of the 10 test results.

### 2.7. Model development and stacking

**Figure 2:**
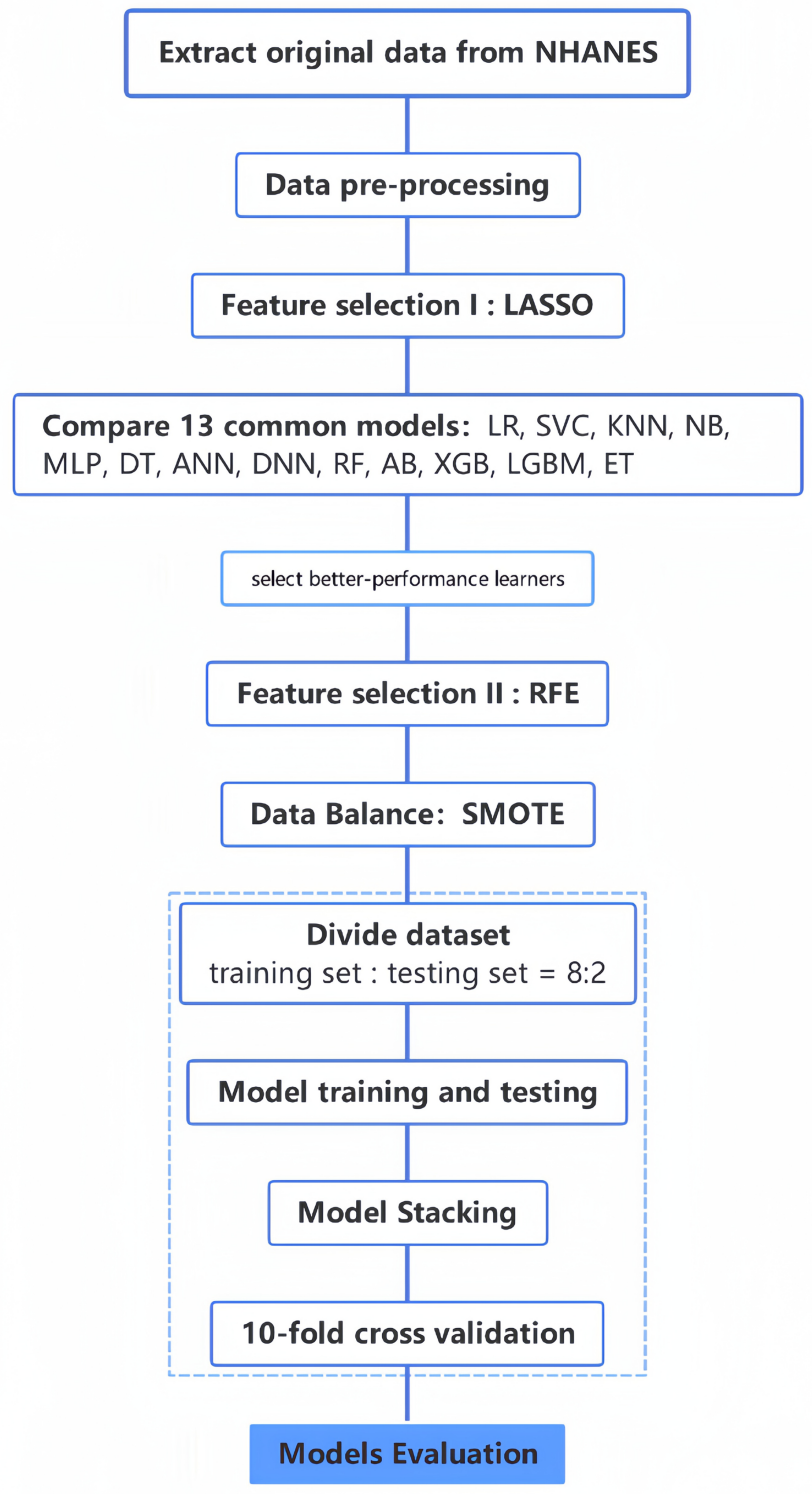
The complete flowchart of this study.

After determining the optimal feature subset, to avoid the noise caused by filling in the missing values, we only retain the complete part of the optimal feature subset in the original data for the construction of the prediction model (34). We used the top four algorithms to build prediction models for only the best subset of features and the dataset with data balance. To make the models more robust, we performed stacking, which is an integrated learning approach that uses another type of learner to perform predictions by using the predicted results of multiple base learners as features (35, 36). Using this approach, different types of base learners can be combined, which can leverage their strengths and improve the performance of the model. We performed stacking based on these four models to obtain a fusion model and compared the performance of this fusion model with the four base models to determine the final prediction model. The flowchart of this study is shown in Figure 2.

### 2.8. Statistics

Statistical analysis used in this study was completed through SPSS 23 and R 4.1.3. For continuous variables, normality was first tested through the Kolmogorov-Smirnov test, means and standard deviations were used if they followed a normal distribution (ANOVA was used to compare continuous variables between groups); if they did not follow a normal distribution, the median (quartiles) were used (non-parametric tests were used to compare continuous variables between groups). For categorical variables, counts and percentages were used (the chi-square test was used to compare categorical data), and all statistical tests were two-sided (*P* < 0.05 was considered statistically significant). Processes such as feature engineering, data balancing and prediction model construction were done in python (Anaconda, Version 3.8), while TensorFlow 2.11.0; scikit-learn 1.1.3; keras 2.11.0; pandas1.5.2; numpy, etc. Package installation is complete.

## 3. Results

### 3.1. Baseline characteristics of the study participants

Of the 8,073 subjects included in NHANES, 50.3% were female and 49.7% were male, and the mean age of the subjects was 50 (35–63) years old.

According to the classification criteria of this study: 3,527 (43.7%) were hypertensive, 892 (11.0%) were pre-hypertensive; 3,654 (45.3%) were normal blood pressure. The mean blood pressure of hypertensive patients was 134 (121–147) mmHg/82 (80–88) mmHg; the mean blood pressure of pre-hypertensives was 129 (122–133) mmHg/79 (71–85) mmHg; the mean blood pressure of normotensives was 113 (106–121) mmHg/70 (65–75) mmHg. More basic information such as BMI, waist circumference, educational status, marital status, physical activity, and smoking status is shown in Supplementary Table S1 in the Appendix.

### 3.2. Analysis of features

**Figure 3:**
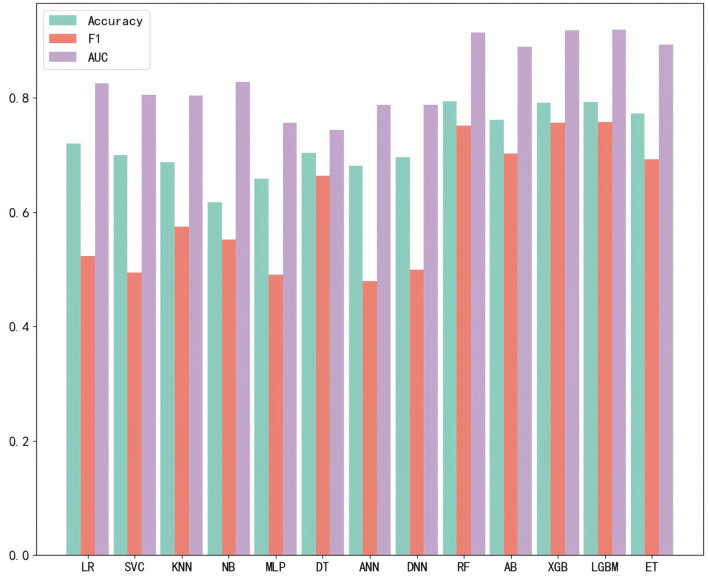
Preliminary model trial.

**Figure 4:**
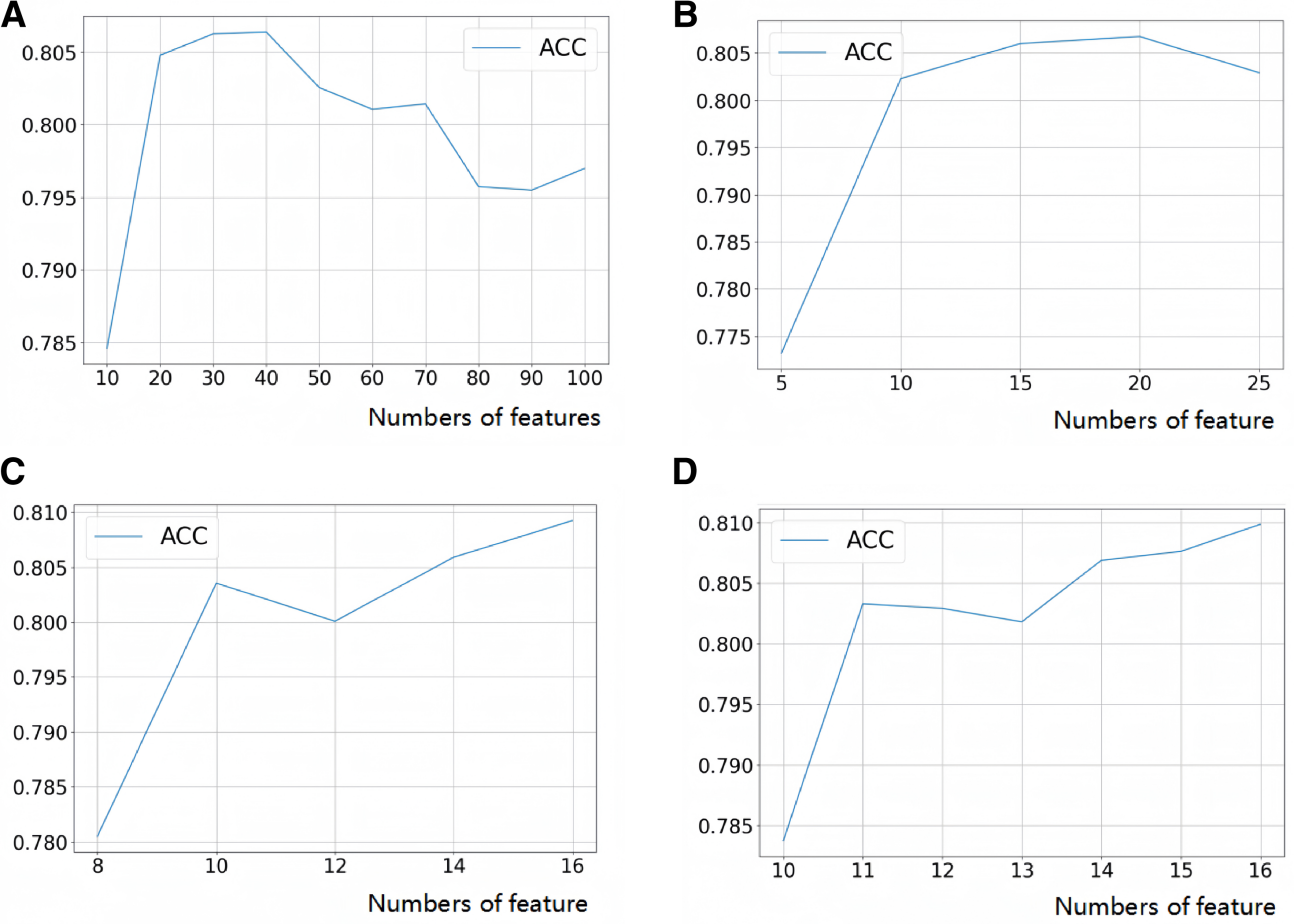
Feature selection process (take random forest as an example). (**A**) Step size = 10, search the range of optimal feature subset; (**B**) step size = 5, search the range of optimal feature subset; (**C**) step size = 2, search the range of optimal feature subset; (**D**) step size = 1 to search for the optimal feature subset.

After LASSO regression analysis, we excluded 12 redundant characteristics: platelet count, total bilirubin, Cotinine, serum cotinine, number of ready-to-eat foods in the last month, number of frozen foods/pizza in the last month, whether or not had asthma, whether or not exercising on the advice of their doctor, whether or not had seen a psychiatrist in the last year, dairy consumption in the last 30 days, weight loss through diet pills/surgery, whether or not have hepatitis B, whether or not had tried to quit smoking. After correlation analysis, we excluded 8 more characteristics: hip circumference, Red cell distribution width, Aspartate Aminotransferase (AST), glucose (frozen serum), Osmotic pressure, total cholesterol (frozen serum), triglycerides (frozen serum), and urinary albumin. After a preliminary model trial, we found that among these thirteen ways the four tree-based classification models, i.e., XGBoost, Extra Tree, Random Forest, and LightGBM, performed better (Figure 3). Therefore, these four base learners were combined with RFE for feature selection. In this process, the accuracy of the model was taken as the measurement target, and the steps of 10, 5, 2 and 1 were taken respectively to gradually determine the optimal feature subsets of different models and RFE combinations. We plotted this process with the number of features as the horizontal coordinate and the corresponding model accuracy as the vertical coordinate, (Figure 4) taking RF as an example. For the feature selection process of other models, please see the Appendix for details.

**Figure 5:**
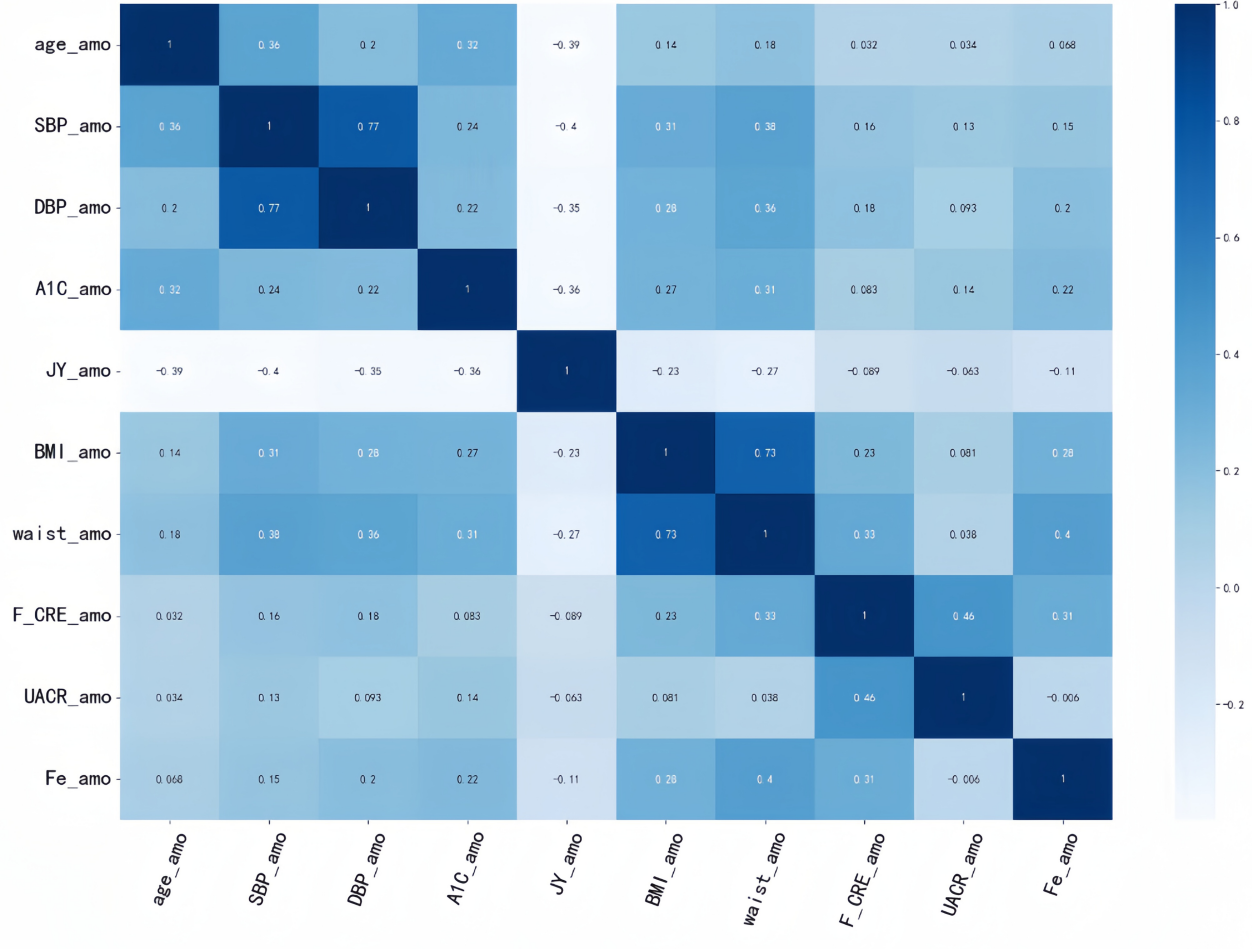
Correlation heat map. It is generated to show the correlation between different features. The correlation strength characteristics before any two features can be shown in this figure. The darker the color, the higher the correlation between the corresponding two features.

**Figure 6:**
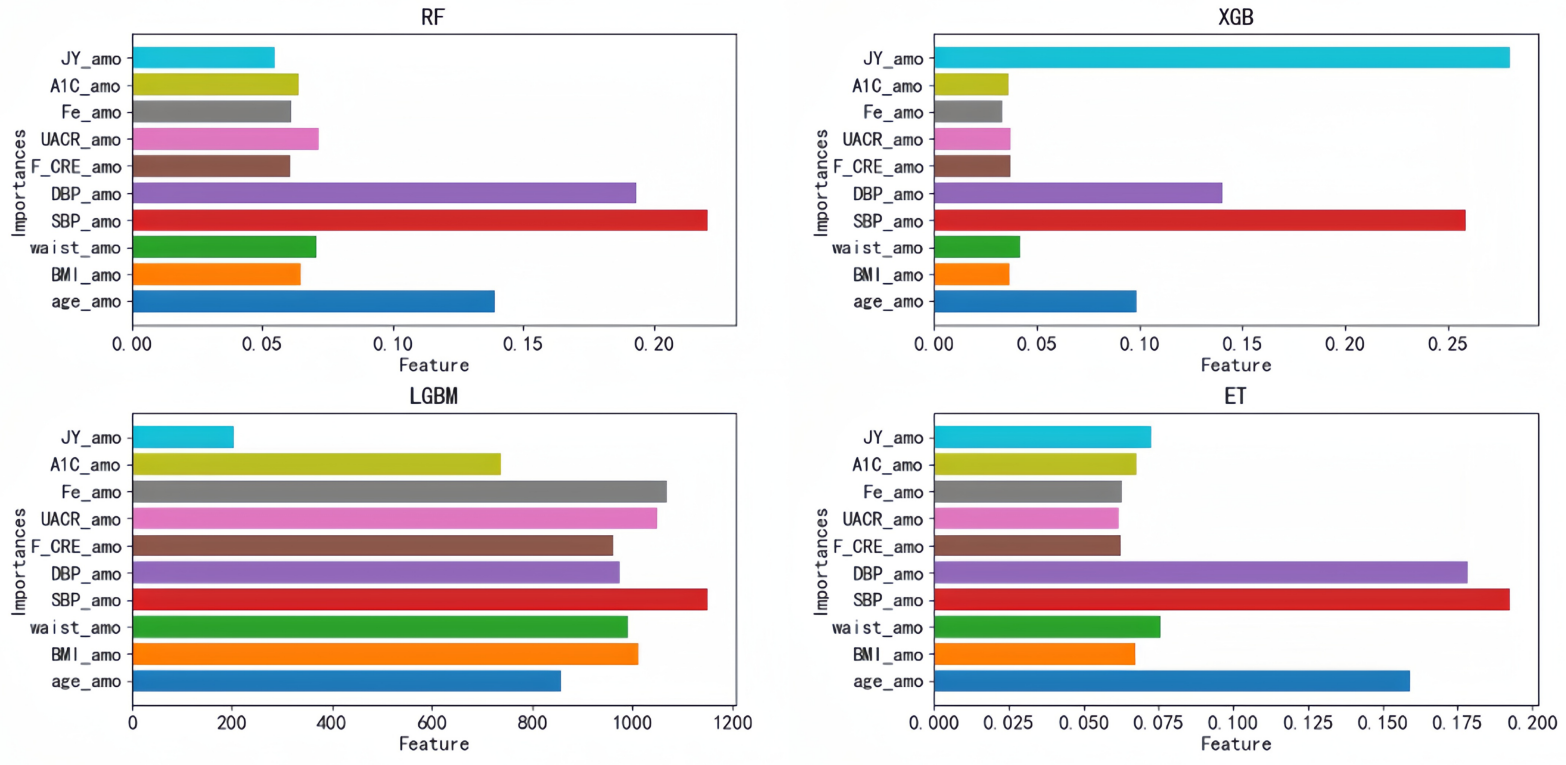
Feature ranking diagram: importance ranking of the optimal feature subset based on the top four models; each bar represents a feature, and the length of the bar represents the importance of the feature to the model prediction results. JY_amo, reduction in salt intake recommended by doctors; A1C_amo, HbA1C glycosylated hemoglobin; Fe_amo, serum ferritin; UACR_amo, urine albumin creatinine ratio; F_CRE_amo, frozen serum creatinine; DBP_amo, diastolic blood pressure; SBP_amo, systolic blood pressure; waist_amo, waist; BMI_amo, BMI; age_amo, age.

Each of these four models has its corresponding optimal feature subset (Table 1). Therefore, features that appear twice or more in these optimal feature subsets are selected to form the final optimal feature subset, that is, age, BMI, waist, SBP, DBP, Cre, UACR, SF, HbA1C, doctors recommend reducing salt intake. Then, we plotted the correlation heat map and feature ranking map to more intuitively show the correlation and ranking between various features (Figures 5, 6).

**Table 1:** The optimal feature subset of different basic learners.

Model	Optimal feature subset
Random Forest	Age; BMI; waist; WHtR; SBP; DBP; F_CRE; UACR; Fe; HbA1C; JY.
XGBoost	Age; SBP; DBP; T2DM; overweight; kinds; JY; JY2; HTC; ReTC; HUQ010; HUQ051; drink0; sleep disorder.
LightGBM	Age; Fe; waist; SBP; DBP; LBXRBCSI; LBXPLTSI; TC; HsCRP; F_CRE;UCR; UACR.
Extra trees	JY; age; BMI; waist; WHR; SBP; DBP;UA; HbA1C.

WHtR, waist-to-height ratio; F_CRE, creatinine, refrigerated serum (umol/L); UACR, albumin creatinine ratio (mg/g); Fe serum, ferritin; HbA1C, glycohemoglobin (%); JY, reduction in salt intake recommended by doctors; T2DM, whether had Type 2 diabetes; overweight Whether overweight or not; kinds how many cardiovascular diseases suffered (including angina, stroke, coronary heart disease, heart failure, heart disease, etc.); JY2 follow doctors’ advice to reduce salt intake; HTC, whether had high cholesterol; ReTC, whether took cholesterol-lowering drugs; HUQ010, general health condition; HUQ050, times receive healthcare over past year; drink0, Ever had a drink of any kind of alcohol (a drink mean a 12 oz. beer, a 5 oz. glass of wine, or one and a half ounces of liquor); sleep disorder, Ever told doctor had trouble sleeping; LBXRBCSI, Red blood cell count (million cells/ul); LBXPLTSI, Platelet count (1,000 cells/ul); TC, total cholesterol (mmol/L); HsCRP, high-sensitivity C-reactive protein (hs-CRP) (mg/L); UCR, creatinine, urine (umol/L); UA, uric acid.

### 3.3. Comparative models

**Figure 7:**
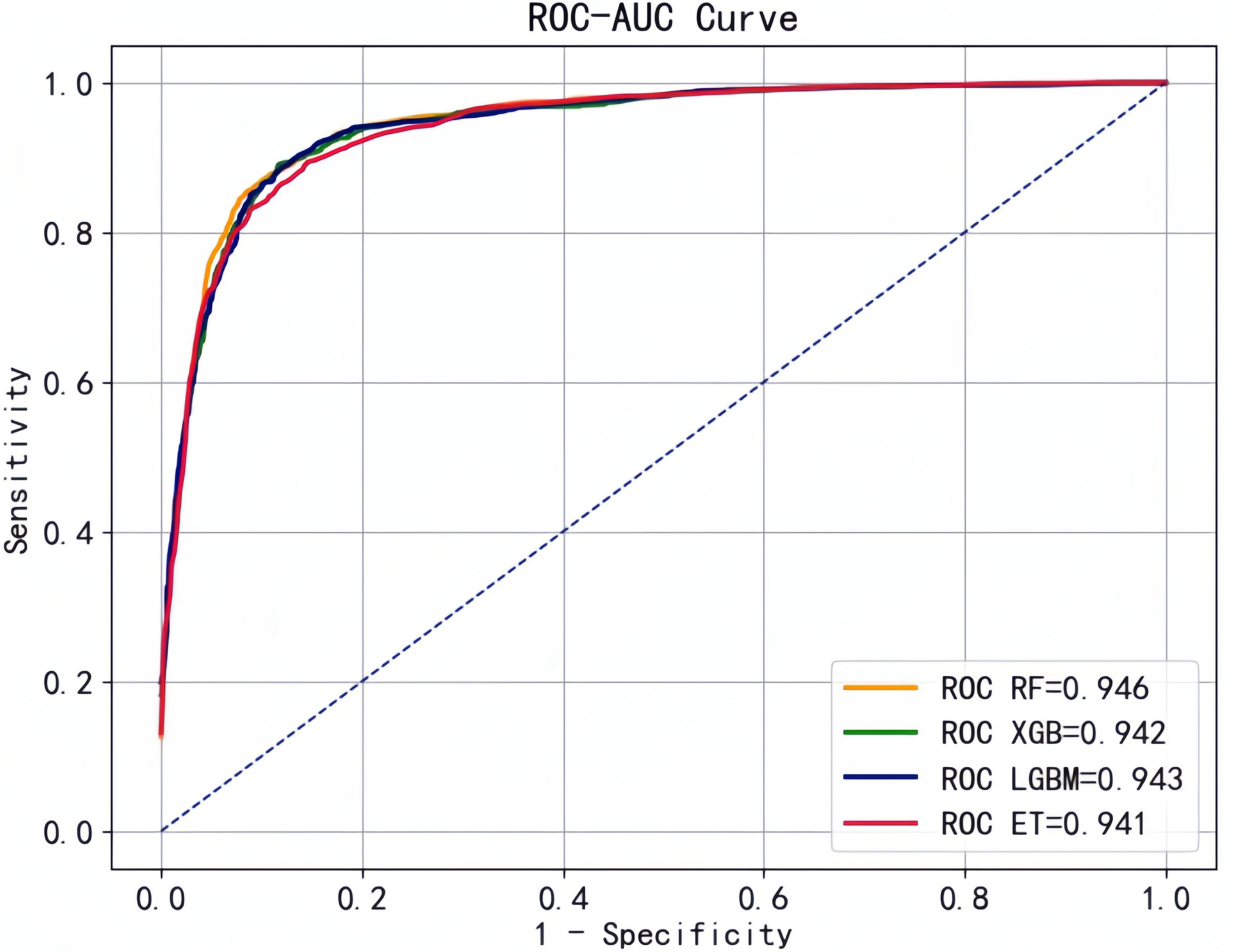
ROC curve of basic learners.

Based on the optimal feature subset , we retained observations (*n* = 5973) with all 10 variables complete in the original data for model prediction and evaluation. Then, we selected RF, XGB, LGBM, and ET with better performance to construct the prediction models. We plotted the ROC curves with "1-specificity" as horizontal coordinate and "sensitivity" as vertical coordinate, and all four classifiers showed good performance with AUC above 0.90 (Figure 7).

Based on these four base learners, we used the stacking strategy for model fusion, and the results for comparison of these models are shown in Table 2. The performance of the fusion model was further improved: accuracy of 0.871, precision of 0.873, recall of 0.871, F1 value of 0.869, and AUC of 0.966.

**Table 2:** Model performance comparison.

Model	Accuracy	Precision	Recall	F1	AUC
RF_test	0.829	0.802	0.811	0.805	0.940
XGB_test	0.826	0.794	0.803	0.797	0.938
LGBM_test	0.816	0.783	0.796	0.788	0.939
ET_test	0.824	0.794	0.780	0.787	0.936
Stacking_test	0.871	0.873	0.871	0.869	0.966

**Figure 8:**
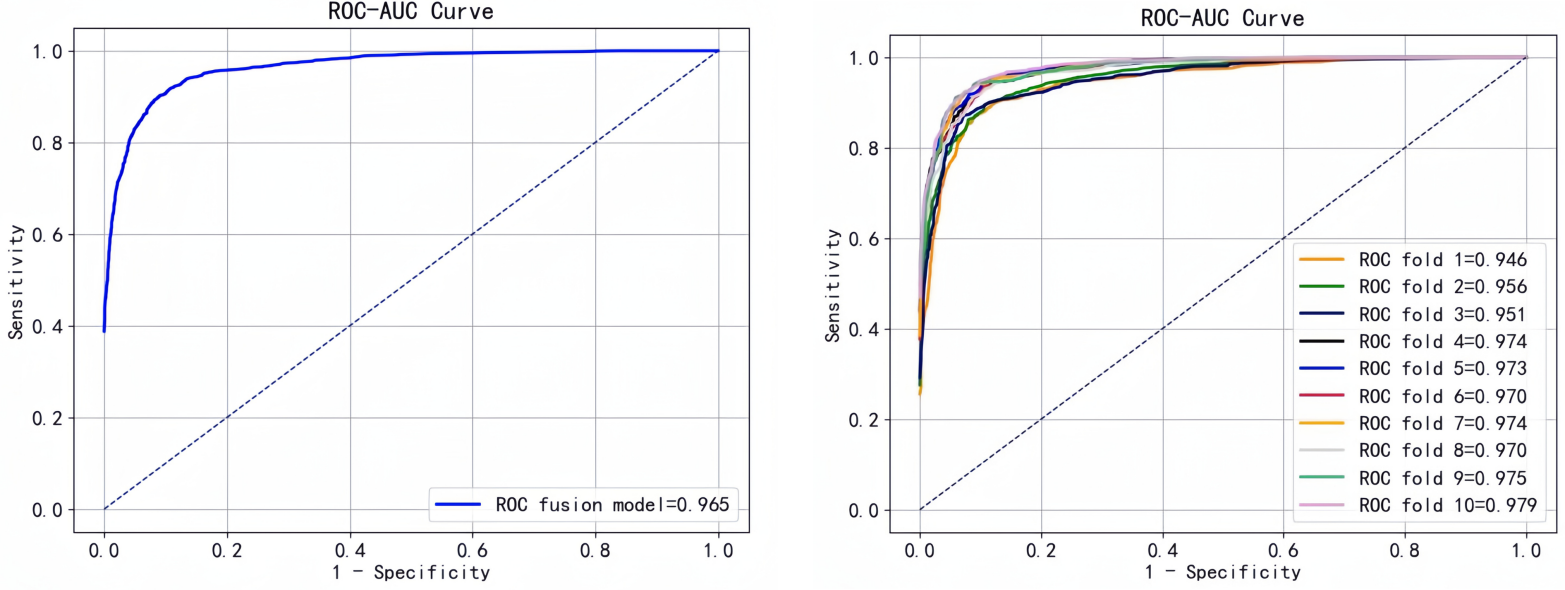
ROC curve of the fusion model and its 10-fold cross-validation ROC curve.

To better evaluate the predictive performance of the model, we performed 10-fold cross-validation of the fusion model and plotted the ROC curve (Figure 8).

### 3.4. Analysis of the association between SF levels and hypertension risk

In this study, we found that SF was included in the optimal feature subset and ranked 8th, 10th, 2nd and 8th in the prediction models constructed by RF, XGB, ET, and LGBM, respectively, which was neglected in the past clinical practice. Therefore, we further analyzed the relationship between SF and hypertension by statistical means.

**Figure 9:**
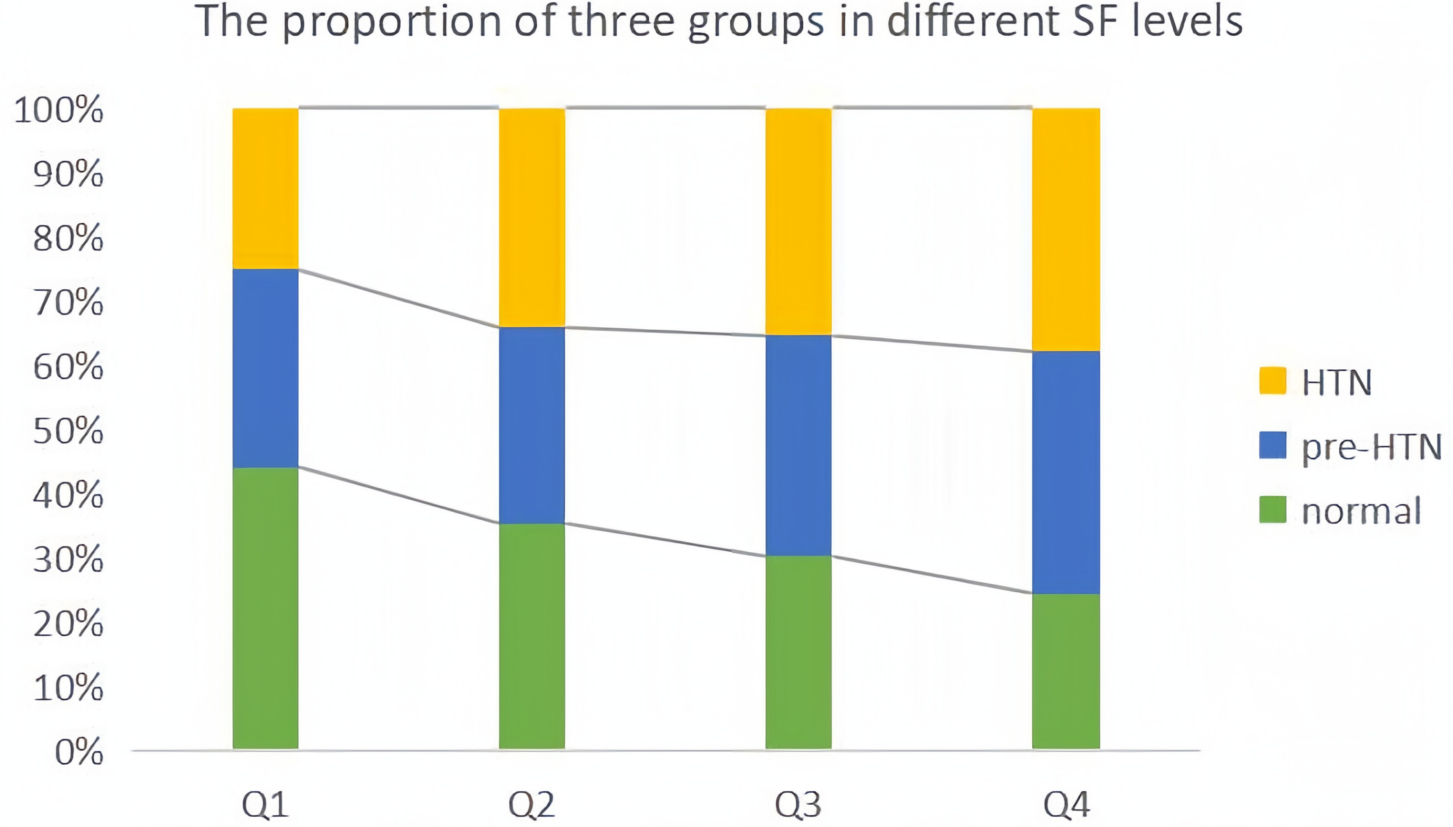
Proportion of three groups in different SF levels.

Since data of SF did not obey normal distribution, we performed the Kruskal-Wallis test and found that the difference in serum ferritin between the three groups was statistically significant (*P* < 0.05), and the mean levels of the three groups are shown in Supplementary Table S1. Meanwhile, we divided the SF data into four levels (Q1-Q4) according to quartiles and plotted the percentage of the three groups in each group (Figure 9). The proportion of hypertensive patients and pre-hypertensives increased step by step, and the proportion of those with normal blood pressure decreased step by step.

We used multiple logistic regression analysis to assess the relationship between SF and HTN with stepwise control for confounding factors. The lowest level group (Q1) was taken as reference, and model 0 was crude. Model 1 controlled for demographic information (sex, age, edu, race, marry, poor); model 2 controlled for anemia and inflammation-related indicators (WBC, RBC, hemoglobin, HsCRP); model 3 controlled for BMI, waist, some potentially confounding medical history (CAD, T2DM, HTC, thyroid disease, COPD, liver disease, kidney disease, etc.) and related metabolic indicators (A1C, TC, HDL, ALT, ALP, CPK, UA, UACR, GGT, etc.); model 4 controlled for smoking, alcohol consumption, diet, sleep, exercise and other lifestyle habits and depression scores; model 5 controlled for factors with significant differences above-mentioned.

Since all models above showed that compared with the lowest level group (Q1), the other three groups (Q2, Q3, Q4) had higher risk, and the OR values with their 95% CI were greater than 1, and the OR values were increasing sequentially with *P *< 0.01. Therefore, we carried out trend analysis for the five models with the help of clm function in R, and the *P* for trend value was <0.001, indicating a trend correlation between the risk of hypertension prevalence and SF levels. The results of the above five models are shown in Table 3.

**Table 3:** Associations of SF with odds of hypertension.

Model type	OR (95% CI)				P_trend_
	Q1	Q2	Q3	Q4	
Model 0	Ref	1.692 (1.482–1.932)	2.054 (1.797–2.348)	2.713 (2.370–3.107)	0.000
Model 1	Ref	1.313 (1.125–1.531)	1.448 (1.235–1.697)	1.692 (1.434–1.997)	0.000
Model 2	Ref	1.936 (1.683–2.227)	2.490 (2.154–2.880)	3.389 (2.913–3.943)	0.000
Model 3	Ref	1.452 (1.240–1.701)	1.560 (1.327–1.834)	1.882 (1.587–2.231)	0.000
Model 4	Ref	1.571 (1.362–1.812)	1.821 (1.576–2.103)	2.276 (1.965–2.637)	0.000
Model 5	Ref	1.396 (1.176–1.658)	1.499 (1.254–1.791)	1.645 (1.360–1.989)	0.000

Model 0: unadjusted.

Model 1: controlling for demographic information (sex, age, edu, race, marry, poor).

Model 2: controlling for anemia and inflammation-related indicators (WBC, RBC, hemoglobin, HsCRP).

Model 3: controlling for BMI, waist, some potentially confounding medical history (CAD, T2DM, HTC, thyroid disease, COPD, liver disease, kidney disease, etc.) and related metabolic indicators (A1C, TC, HDL, ALT, ALP, CPK, UA, UACR, GGT, etc.).

Model 4: controlling for smoking, alcohol consumption, diet, sleep, exercise and other lifestyle habits and depression scores.

Model 5: controlling for factors with significant differences above-mentioned.

OR, odd ratio; CI, confidence interval; Q, quartile.

P_trend_, *P*-values for trend analysis of the relationship between SF and hypertension.

## 4. Discussion

Our study proposes a high-performance and convenient model for predicting the risk of hypertension, which can be used as an automated diagnostic aid in a hospital environment in conjunction with EMR data for real-time prediction.

In the preliminary screening stage of the model, we used 13 methods to construct the prediction model in parallel and used ACC, AUC and F1 as the measures, and found that four decision tree type methods, RF, XGB, LGBM and ET, performed better. We consider that it may be because the decision tree type methods have advantages in dealing with high-dimensional data and nonlinear relationships, and can better capture patterns in data features, which are more suitable for the data features and prediction task requirements of this study. In addition, the fusion model constructed based on the stacking strategy also has better stability and can be more reliably applied in clinical practice.

The single measurement of SBP and DBP fluctuates rapidly under the influence of various factors such as mood, sleep, exercise and time. However, our proposed prediction model incorporates laboratory tests with relatively stable changes. For example, HbA1C is used to assess the average level of blood glucose levels over the past 2 to 3 months; creatinine ratio is used to reflect changes in kidney function and muscle metabolism, it always changes little in people with normal kidney function, but changes within a month in people with kidney disease; serum creatinine is used to reflect changes in filtration function of the kidney, it only changes dramatically in days in patients with acute kidney injury and significantly in months or years in patients with chronic kidney disease; fluctuations in SF are generally not significant, usually taking weeks or months, and for the treatment of anemic patients, changes in this index also take 1 to 3 months, unless the patient experiences acute inflammation, which can change dramatically in a short period of time. In a short, such a predictive model is more stable compared to office blood pressure measurements. The included laboratory data are valid for at least 1 month. Also, multiple inputs can be obtained from HIS (Hospital Information System), it is more cost-effective than ambulatory blood pressure. We considered that it could be used as an early warning signal to reduce admission rate, readmission rate and their expenses, avoiding progression to more serious disease (37, 38). It can also be used for mass screening in communities, helping communities achieve level zero prevention at a lower cost (39–42).

In addition, hypertension is a multi-factorial disease, and many factors such as age, BMI, smoking, alcohol consumption, and excessive salt consumption are widely recognized by clinical experts as major influencing factors. Therefore, most studies on hypertension risk prediction have focused on these factors. Our study, based on data from NHANES 2017 to March 2020, incorporated more comprehensive information. We considered the optimal subset of features selected, and with the exception of serum ferritin, the other 9 features have been extensively documented to correlate with hypertension, especially factors such as age and BMI have been widely accepted as influencing factors in the clinic. SF has been less studied in the field of hypertension, so we decided to further explore the relationship between SF and hypertension.

SF is a recognized biomarker used to evaluate the status of iron (iron deficiency or iron loading) (43, 44) and its levels vary greatly by race and gender. In men, SF levels start rising in adulthood, peaking between the age of 30 and 39 years, and then remain relatively stable. In women, it starts rising only at menopause (around 50 years of age) and reaches a plateau around 60 years. Adult Black men generally have higher average SF levels than adult White men (45, 46).

The SF levels can increase significantly due to inflammation or the occurrence of certain diseases (liver or kidney disease, malignancy, metabolic syndrome, etc.) and play an important role in energy metabolism disorders (45). For example, ferritin levels were found to be correlated with interleukin-6 and hypersensitive c levels (47). Metabolic disorders were found to be related to elevated iron stores in the Chinese population (44). The reduction in ferritin levels to the levels found in children and premenopausal women can significantly improve clinical outcomes (the primary outcome is death due to all causes, and secondary outcomes include non-fatal myocardial infarction and stroke) (47).

By searching several databases, we found that very few studies investigated the relationship between SF and hypertension. Considering that high levels of SF are a characteristic of insulin resistance-associated hepatic iron overload syndrome (IRHIO), Piperno et al. (48) divided the study population into primary hypertension patients, IRHIO patients, and individuals with normal blood pressure and found that SF was higher more often in hypertensive men than in the participants in the control group (47). Mee Kyoung et al. followed 8,580 men without hypertension at baseline for four years and compared the baseline SF levels in 818 men who finally developed the disease with that of the remaining 7,000 (approx.) men with normal blood pressure and found that these 818 men had significantly higher SF levels than the others (49). Jae-Hong et al. studied 7,104 healthy men enrolled in KNHANES-2005 (a medical health screening program for Koreans) and divided them into four groups based on the baseline SF levels. The participants were followed for five years, and the incidence of hypertension was compared among the four groups of participants; the group with the lowest incidence of hypertension was considered to be the control group. The researchers found that the adjusted hazard ratio (HR) and 95% CI of the four groups were 1.00 (control), 1.09 (0.91–1.30), 1.21 (1.01–1.45), and 1.28 (1.07–1.52), respectively (50). Singh et al. conducted a hospital-based observational and analytical study, which included 51 hypertensive patients and 51 healthy individuals, and statistically compared the two groups for various indicators: SF (293.27 ± 219.84 ng/ml, 72.23 ± 29.75 ng/ml), mean systolic blood pressure (151.45 ± 14.77 mm/Hg, 109.88 ± 5.43 mm/Hg), and mean diastolic blood pressure (95.56 ± 7.46 mm/Hg, 72.43 ± 2.97 mm/Hg). The mean SF levels and hypertension in hypertensive patients were positively correlated (51).

These four studies either confirmed significant differences in SF between hypertensive and control groups with only a small number of participants, or found that baseline SF levels were significantly higher in hypertensive patients than in normal participants through follow-up, but did not fully account for more relevant confounding factors. We comprehensively considered all the factors that may affect hypertension and SF, and included them as co-variables, and objectively investigated the correlation between SF and hypertension.

Although SF is an inexpensive and widely used biomarker, it has not been used to extensively study its association with hypertension or its complications related to cardiovascular disease and is less commonly included in routine physical examinations.

Of course, our study has several shortcomings. (1) This was a cross-sectional study that did not take into account the long-term development and progression of the disease. (2) Our study is only based on NHANES database, and its representation is limited to the US population. (3) The prediction model constructed in this study only predicted the end event, without further study on disease progression, such as how long it will take to develop the disease. (4) In this study, the relationship between hypertension and SF needs to be further explored on the temporal relationship.

Although the results of this study are limited to the US population, we believe that the methodology proposed in this study is transferable, and we are currently conducting similar studies on Chinese populations to further validate the findings of this paper. In future studies, we will verify the usability of the prediction model constructed in this study on a larger data set based on real hospital data or in cooperation with multiple medical institutions by means of multi-center cooperation, and in this process, we will also pay more attention to sample balance. At the same time, we hope to further confirm the relationship between SF and hypertension using multiple datasets from different sources as well as explore the causal relationship between the two through cohort studies and other means.

## 5. Conclusion

In this study, we propose a cost-effective hypertension risk prediction model based on the stacking strategy that integrates demographic, physical measurements, and physiological and biochemical indicators, etc. to incorporate more comprehensive information. Further statistical analysis of the SF suggested by the optimal subset of characteristics was also performed, and showed that serum ferritin level was trending correlated with the risk of hypertension. In the future, we aim to collect more targeted data and construct more robust predictive models to assist clinicians in diagnosis and increase patient awareness of the disease.

## Data Availability

The original contributions presented in the study are included in the article/**Supplementary Material**, further inquiries can be directed to the corresponding authors.

## Supplementary material

Supplementary Images 1–3 XGBoost and RFE are combined for feature selection, ET and RFE are combined for feature selection, LGBM and RFE are combined for feature selection.
