# Supplementary figures and images for: Development of a convenient and effective hypertension risk prediction model and exploration of the relationship between Serum Ferritin and Hypertension Risk: a study based on NHANES 2017—March 2020
Source: Front Cardiovasc Med. 2023 Sep 6;10:1224795. doi: 10.3389/fcvm.2023.1224795 (PMC10510409; doi:10.3389/fcvm.2023.1224795)

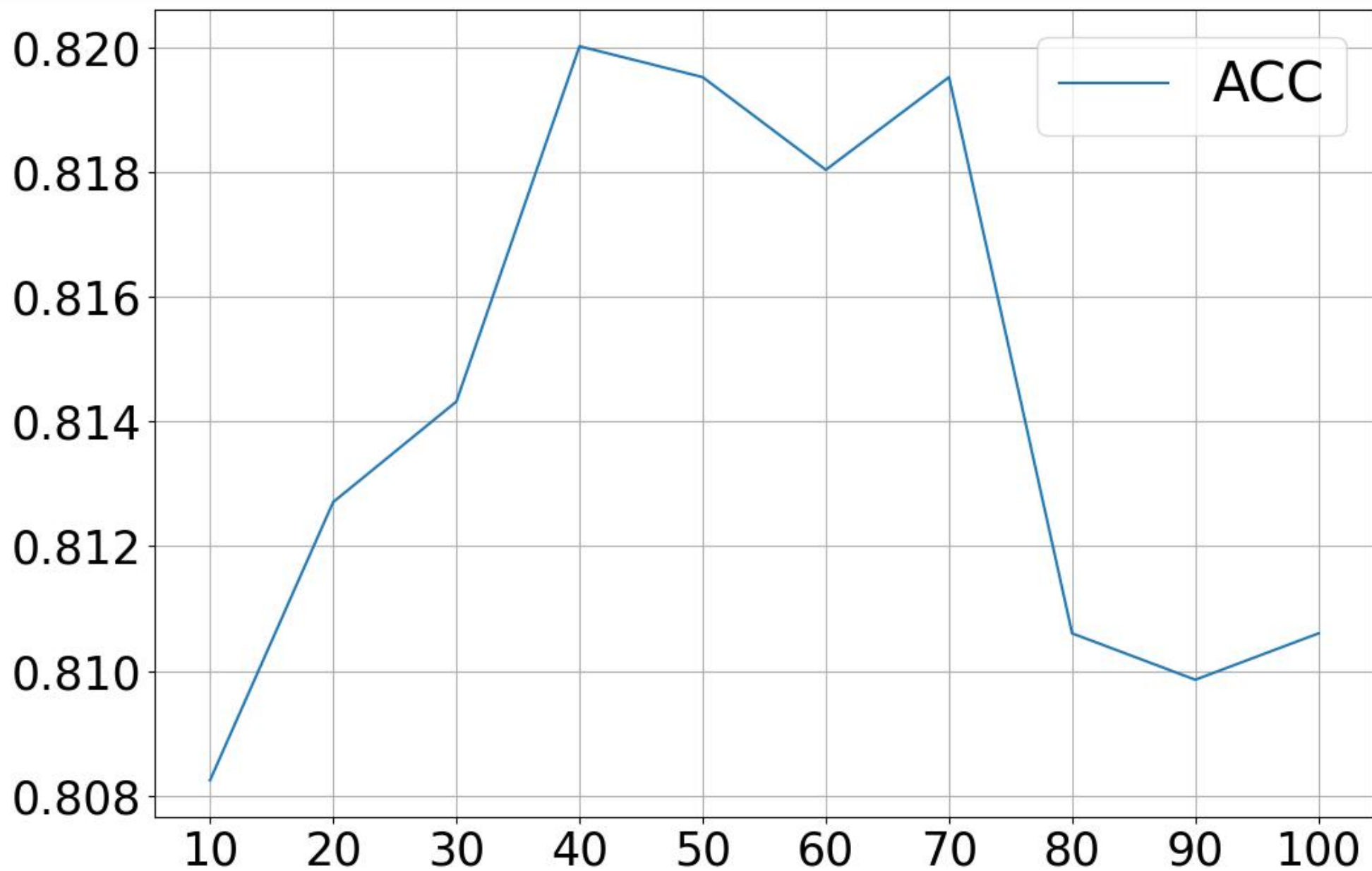

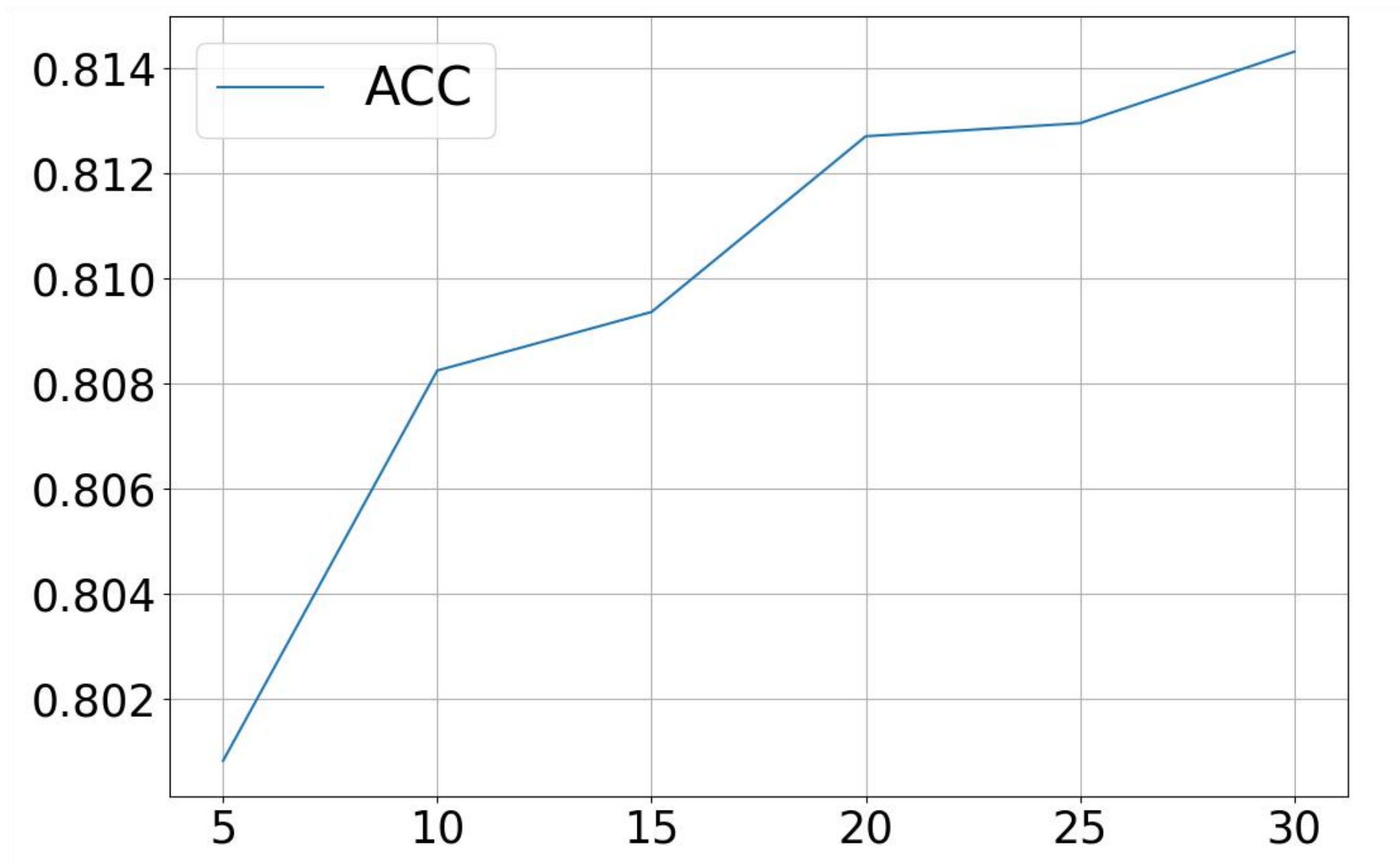

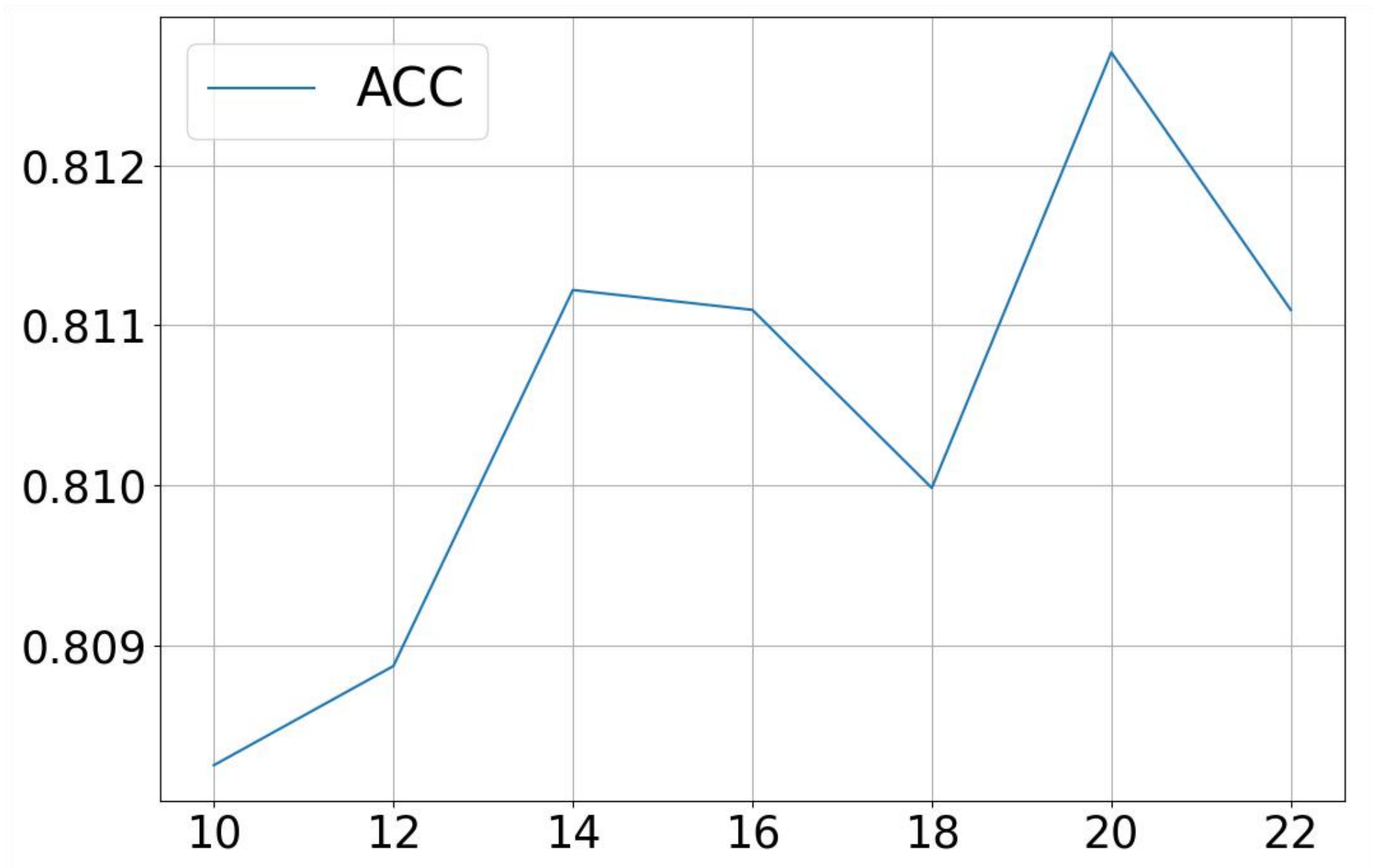

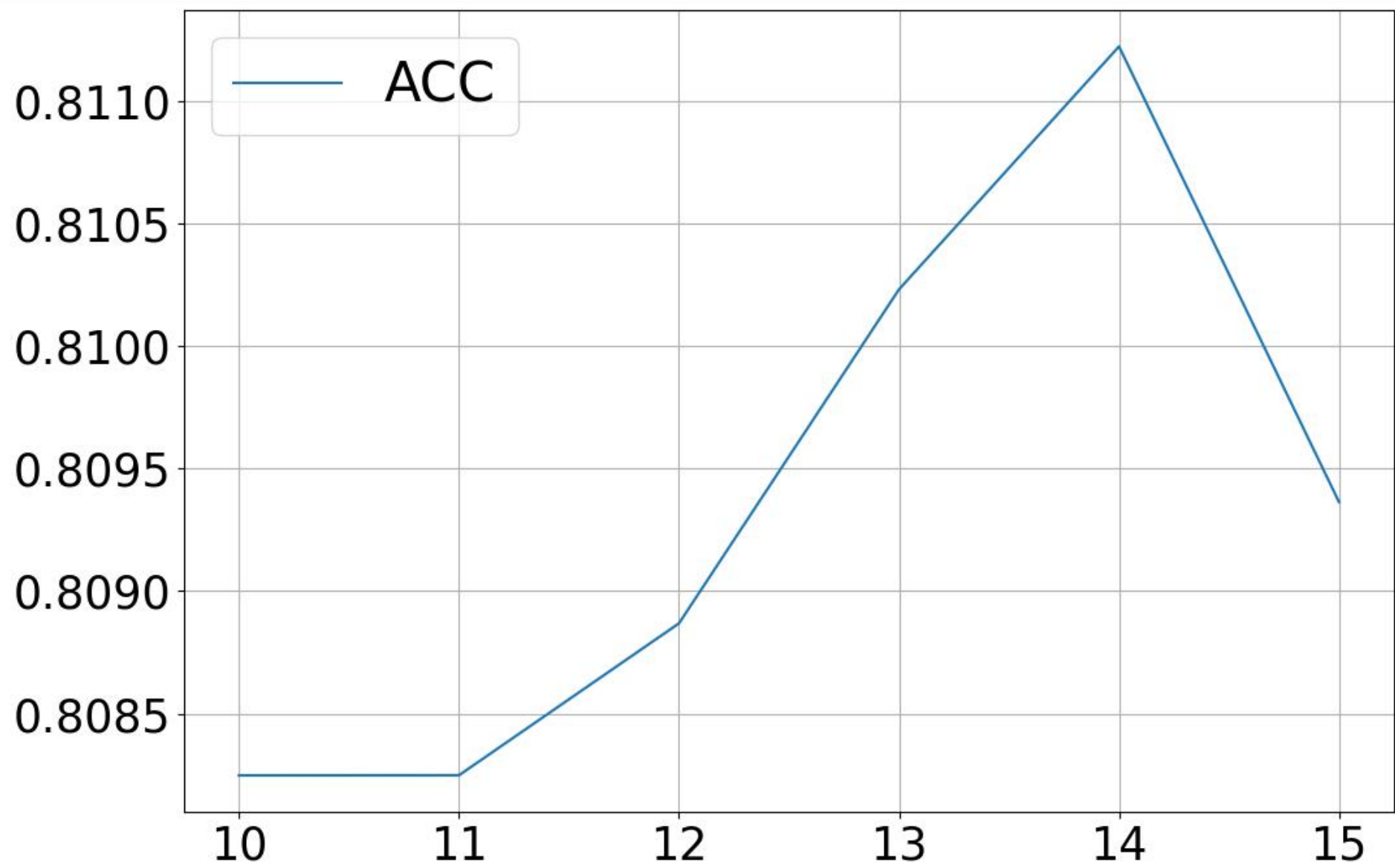

Supplement: Supplementary Images 1–3 — XGBoost and RFE are combined for feature selection, ET and RFE are combined for feature selection, LGBM and RFE are combined for feature selection. [file Image1.pdf]

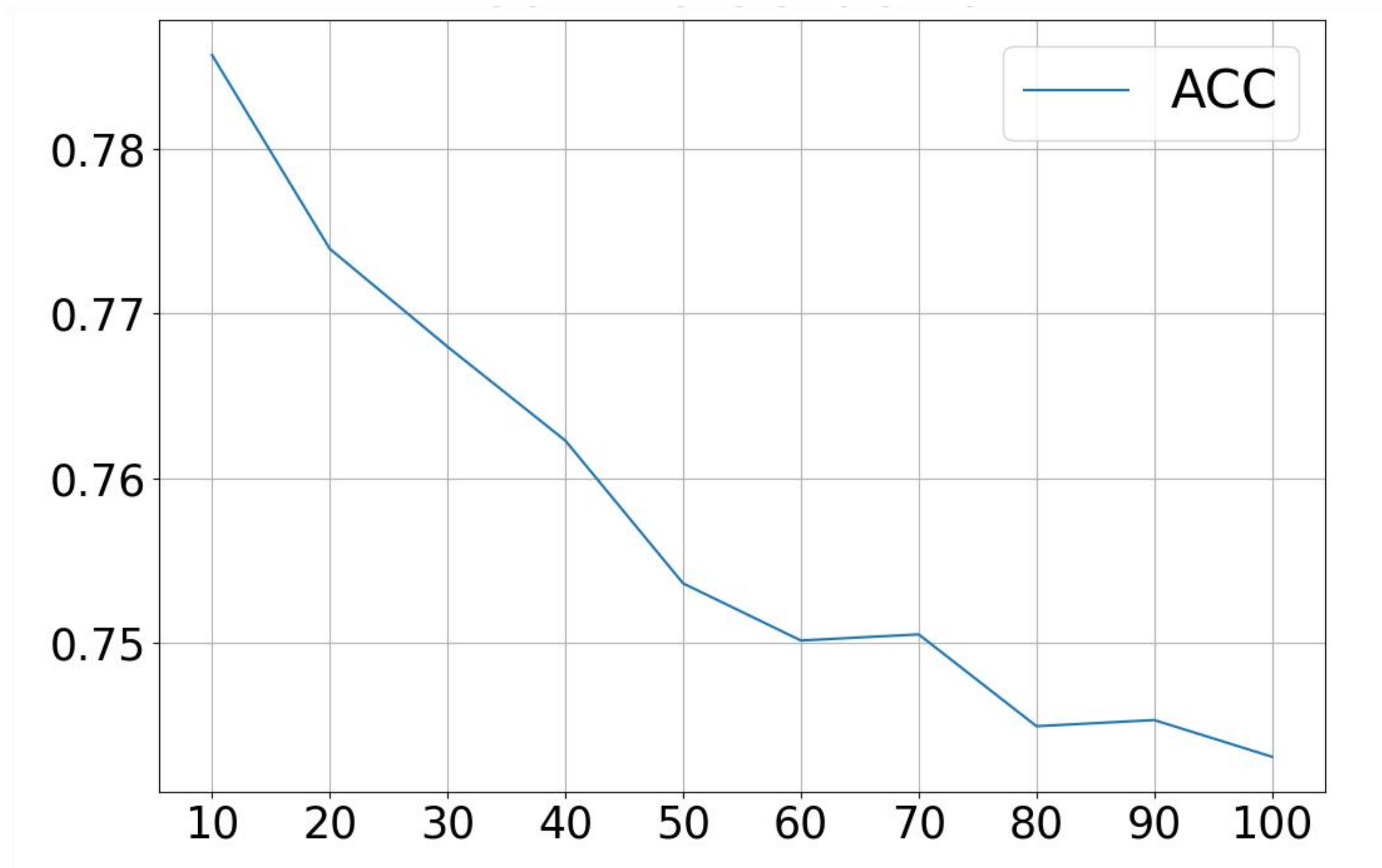

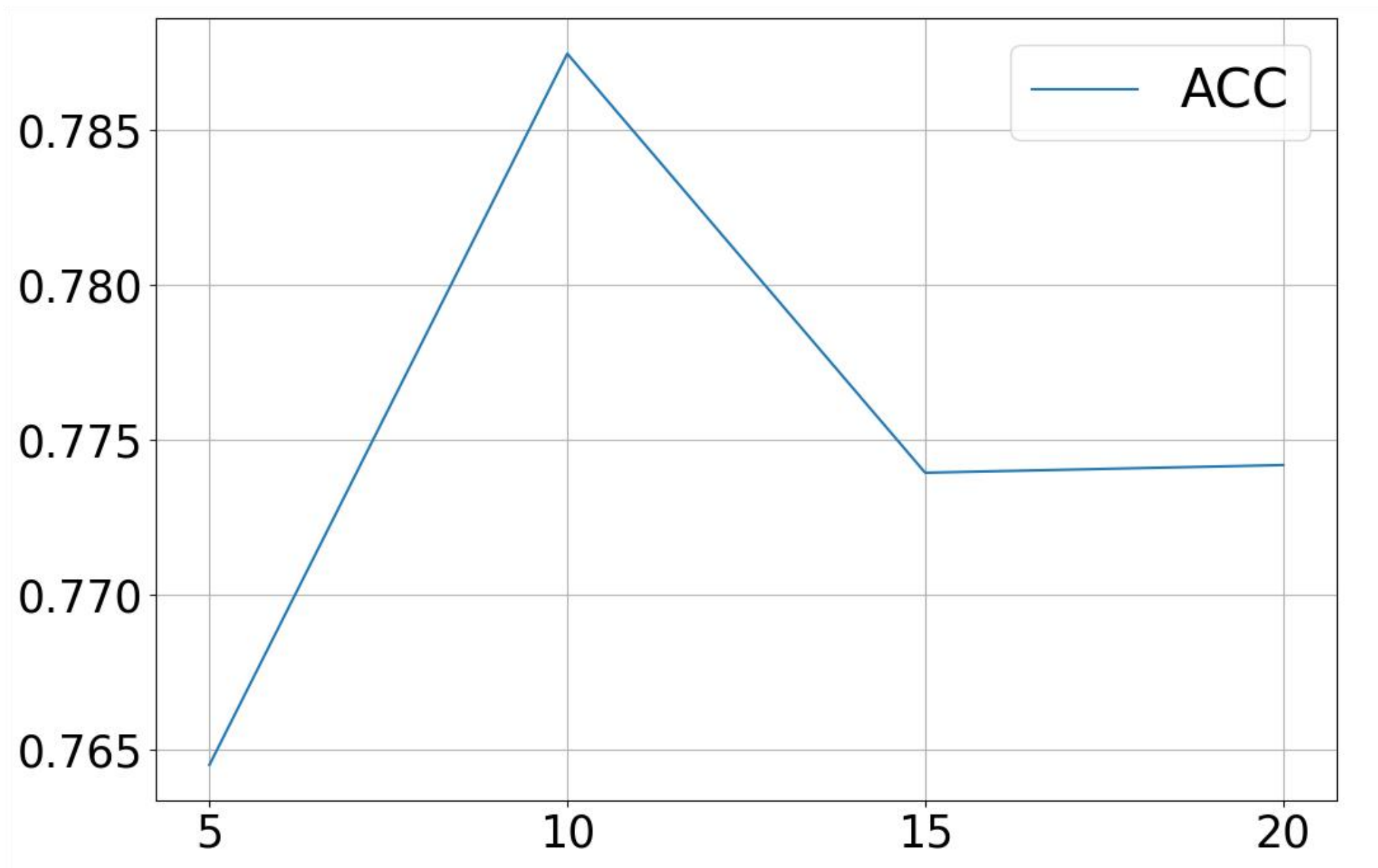

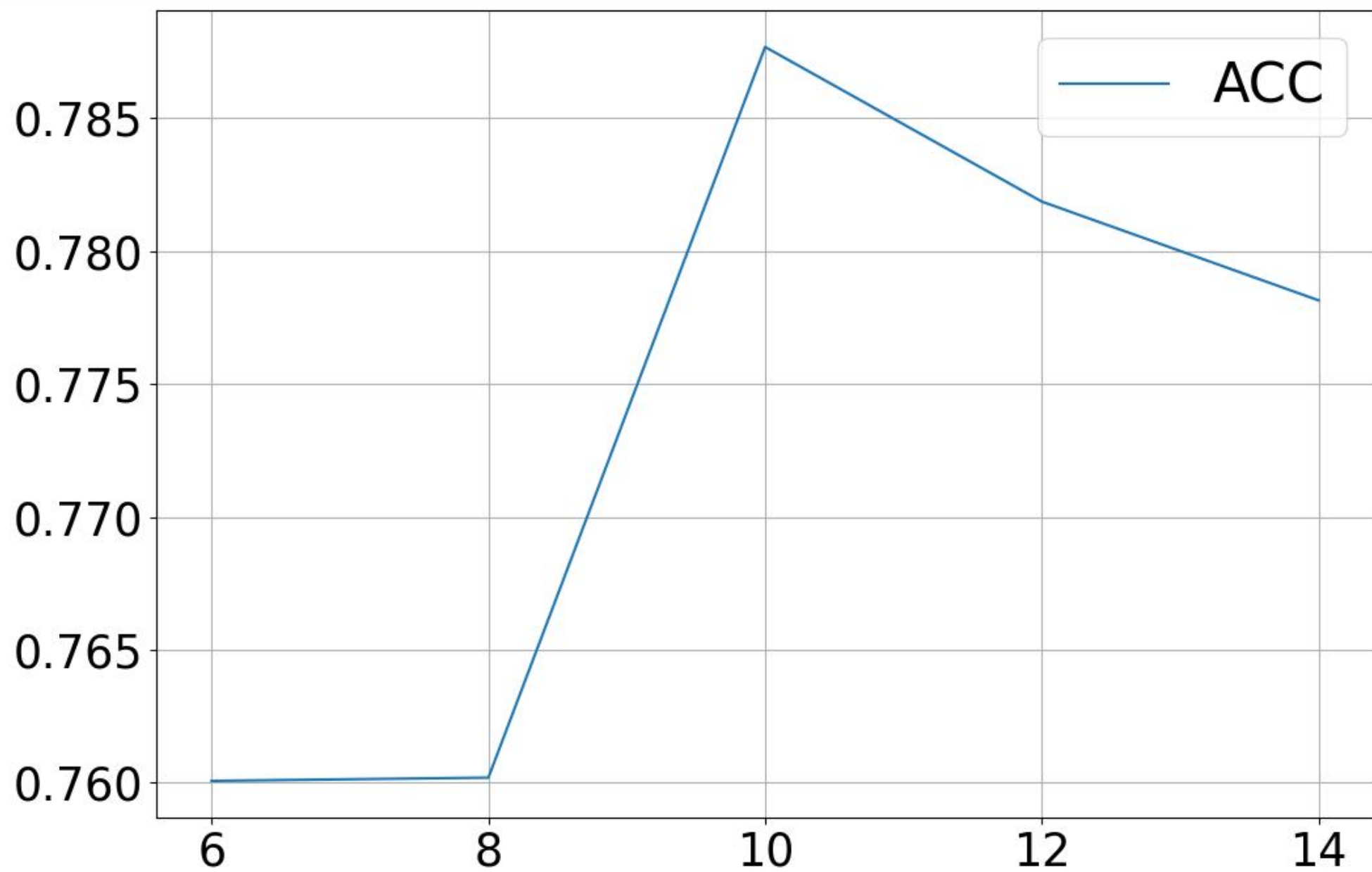

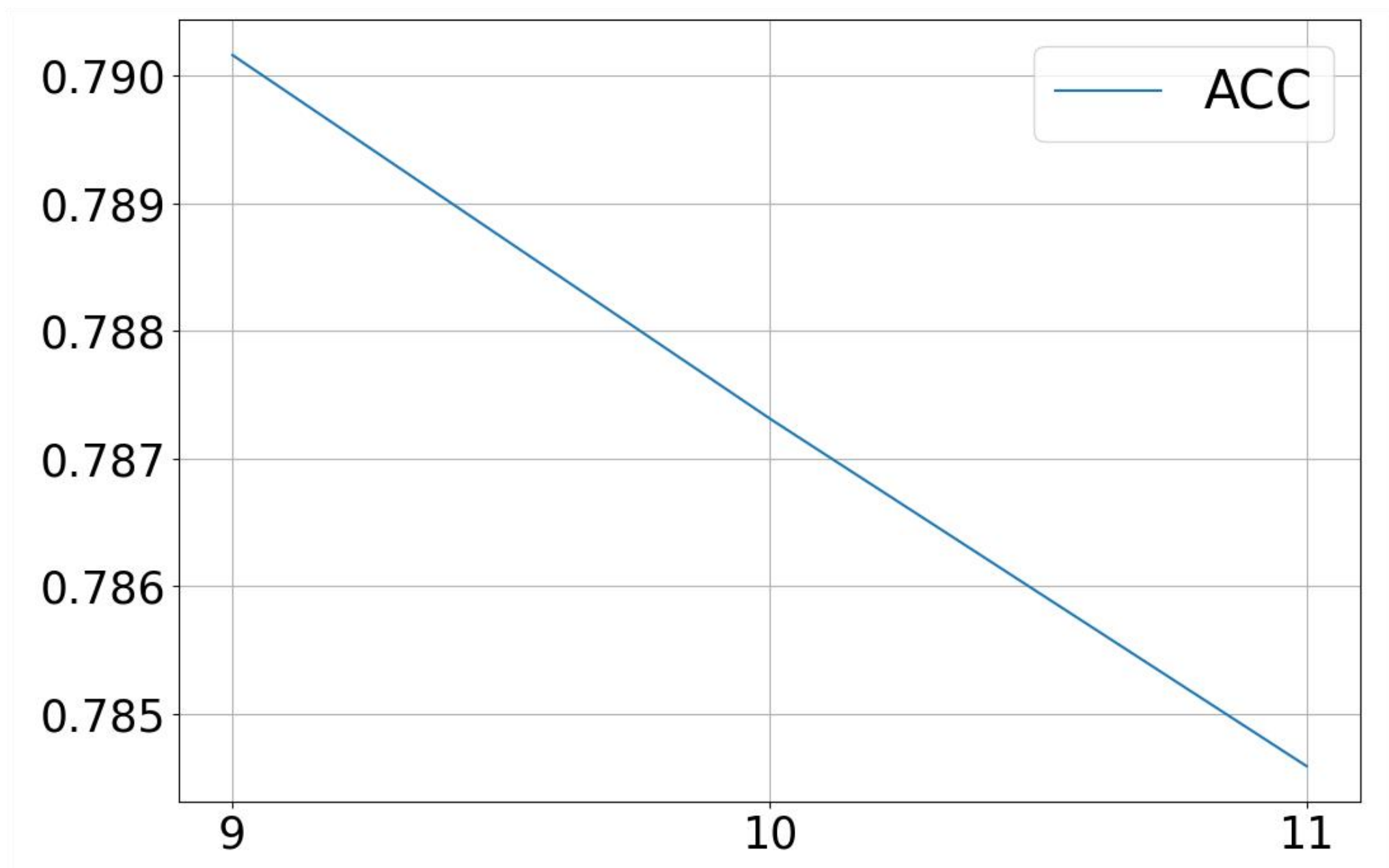

Supplement: Supplementary file 3 [file Image2.pdf]

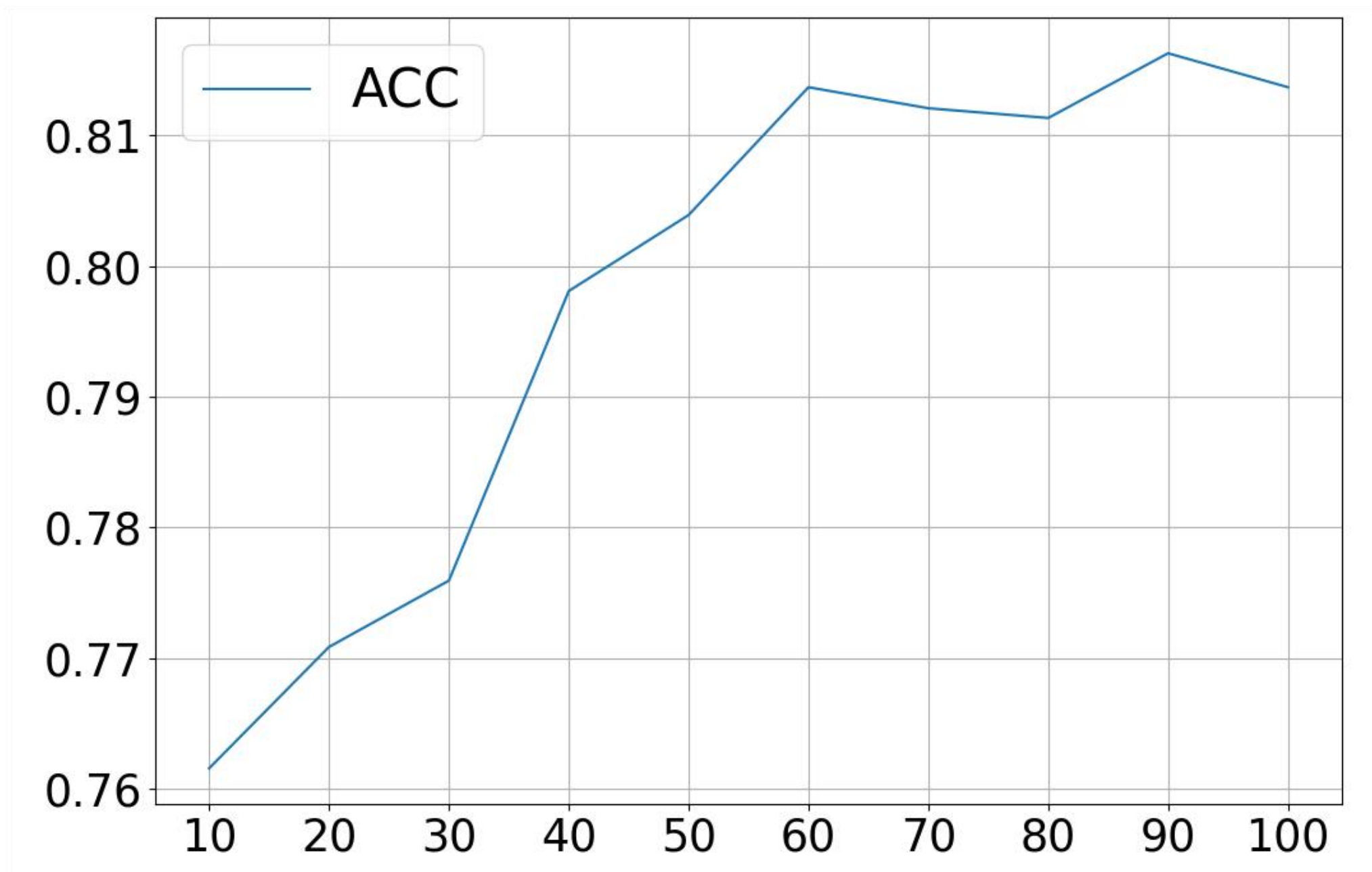

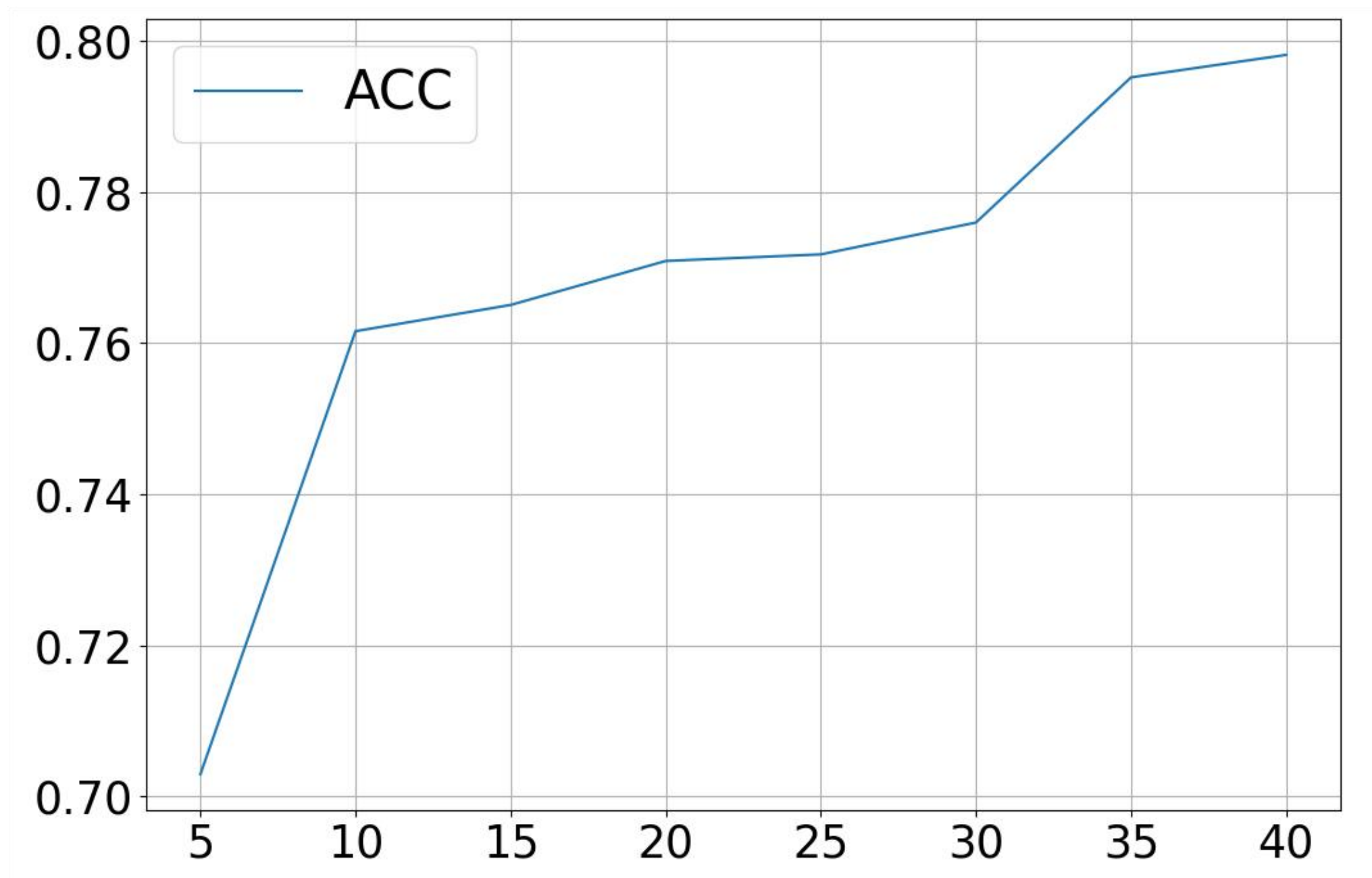

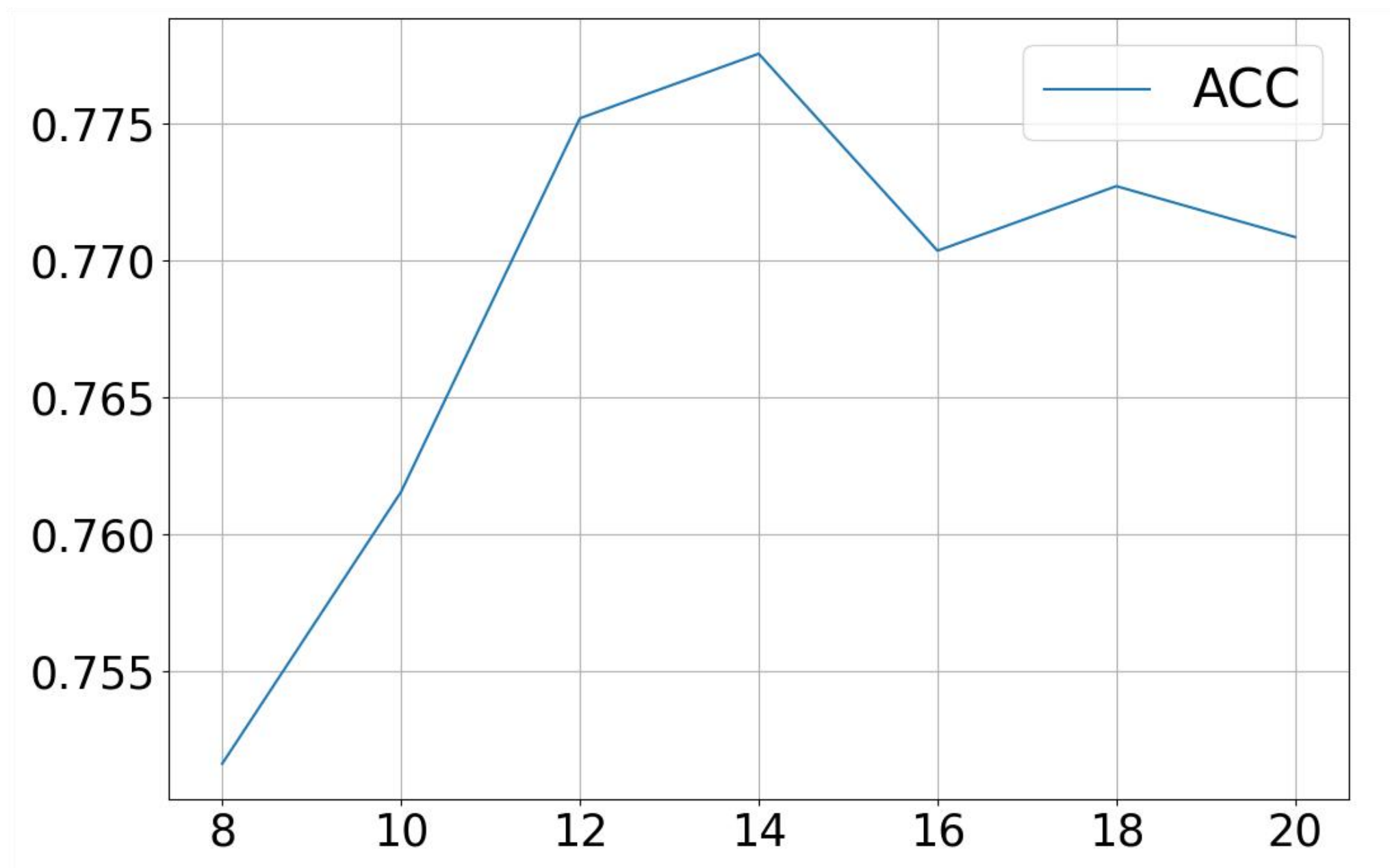

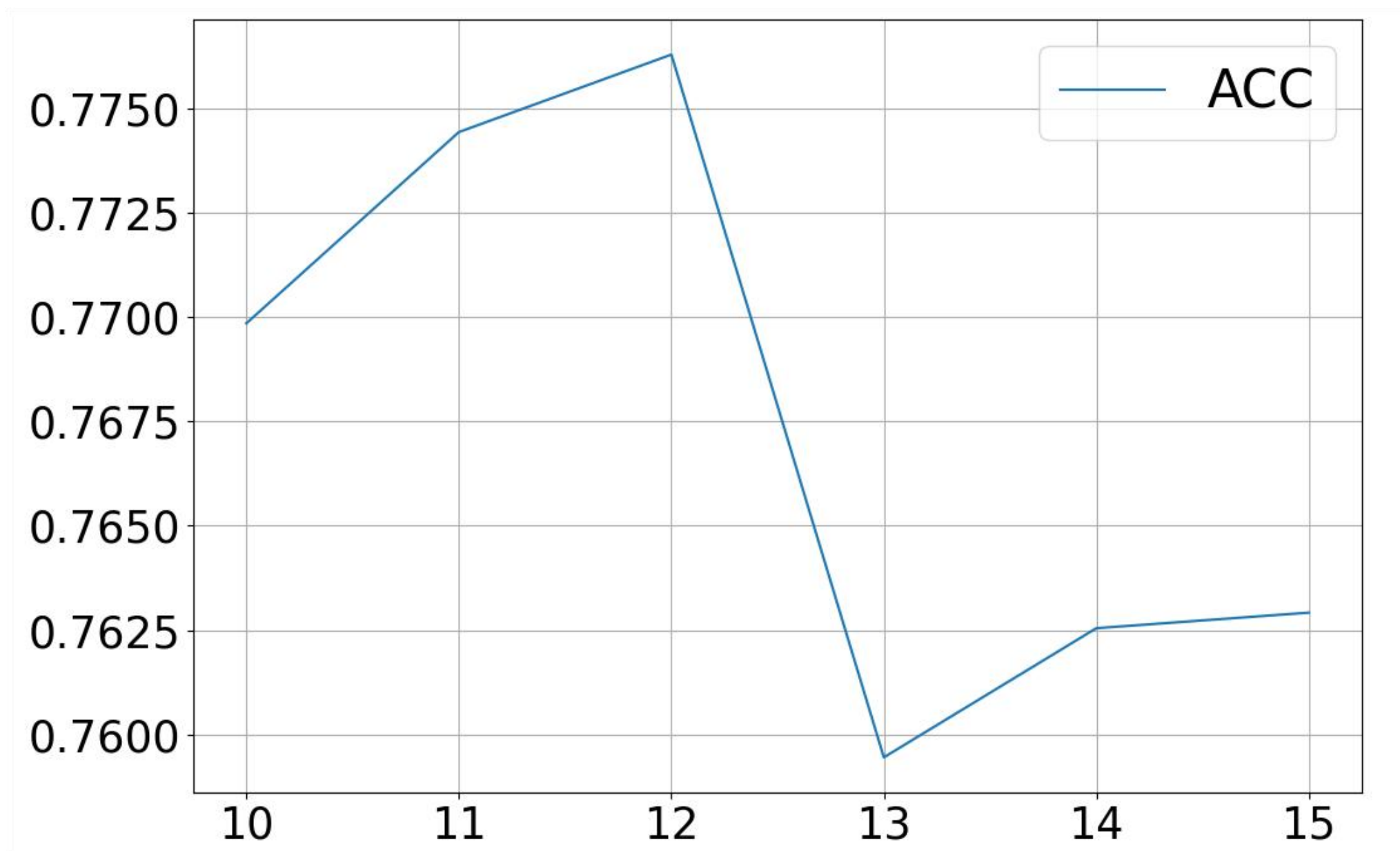

Supplement: Supplementary file 4 [file Image3.pdf]
